# Web Pages on Mindfulness-Based Interventions: A Review on the Different Training of Third-Wave Psychotherapies Available in the United Kingdom

**DOI:** 10.1192/bjo.2022.243

**Published:** 2022-06-20

**Authors:** Jiann Lin Loo, Jashan Selvakumar, May Honey Ohn, Asha Dhandapani, Sathyan Soundararajan, Sahar Ali, Nikhil Gaurishankar

**Affiliations:** 1 Betsi Cadwaladr University Health Board, Wrexham, United Kingdom; 2 St George's University of London, London, United Kingdom.; 3 Croydon University Hospital, London, United Kingdom; 4 Keele University School of Medicine, Keele, United Kingdom

## Abstract

**Aims:**

With extensive evidence and track record on efficiency, third-wave psychotherapies, i.e. mindfulness-based interventions (MBIs), have gained popularity in the United Kingdom (UK) as the mainstream tool for mental health and well-being. During the COVID-19 pandemic, a lot of MBI training has shifted from physical meetings to online to improve access nationally. To date, there is limited data on the differences of online MBIs available in the UK. This web pages review is aimed to elucidate the available resources for online training on MBIs in the UK.

**Methods:**

Google Search engine was used to identify web pages providing MBI training in the UK from February 2021 to March 2021. The search words used were “mindfulness”, “acceptance commitment therapy”, “dialectical behaviour therapy”, “DBT”, “Compassion focused therapy”, “CFT”, “England”, “Northern Ireland”, “Scotland”, “Wales”, and “United Kingdom”. The search word “ACT” was omitted due to a high number of irrelevant search results. Inclusion criteria were any web page providing mindfulness training in the English language, based in the UK. Exclusion criteria were web pages that were not from the UK with limited information and the web page was not about the provision of mindfulness training. Given the high number of web pages appearing in the Google Search for each of the localities, further search was stopped when all ten web pages that appeared on a Google search page were all excluded.

**Results:**

The total number of web pages returned from searches was 23,030,000 of which were 13.1 million for England, 2.89 million for Scotland, 3.09 million for Wales, 2.18 million for Northern Ireland, and 1,770,000 were unspecified. Only 165 web pages offering MBI training were included. Among those, 57% were for the general public while 30% had information for both professionals and the public. The majority of them, i.e. 65% offered online training courses when only 25% of them offered both online and face-to-face training. There were 25% of web pages offering free basic courses for the public. There was a similar split between the group, individual and mixed training.

**Conclusion:**

There is a significant amount of MBI training resources available online for both public and professionals. One interesting finding is that a significant portion of them provide free basic training which is very encouraging and certainly has a positive impact on the accessibility of mindfulness education during the pandemic disruption.

